# The COVID-19 Pandemic and Mental Well-Being of Pregnant Women in Japan: Need for Economic and Social Policy Interventions

**DOI:** 10.1017/dmp.2020.334

**Published:** 2020-09-10

**Authors:** Midori Matsushima, Hanna Horiguchi

**Affiliations:** University of Tsukuba, Faculty of Humanities and Social Sciences, Tsukuba, Japan; Kobe University, School of Medicine Faculty of Health Sciences, Kobe, Japan

**Keywords:** COVID-19, Edinburgh Postnatal Depression Scale (EPDS), Japan, perceived risk, pregnant women

## Abstract

**Objective:**

This study explores the mental well-being of pregnant women in Japan during the coronavirus disease (COVID-19) pandemic.

**Methods:**

We collected 1777 responses from pregnant women through an online survey. Using the Japanese version of the Edinburgh Postnatal Depression Scale (EPDS), we calculated the percentage of pregnant women above the cutoff (≥ 13), and the factor scores of anhedonia, anxiety, and depression. Regression analyses were performed to identify factors and socioeconomic characteristics correlated with depressive symptoms.

**Results:**

The point prevalence of pregnant women with an EPDS score of ≥ 13 was 17%. The mean scores were 0.73, 3.68, and 1.82 for anhedonia, anxiety, and depression, respectively. The probability of becoming above the cutoff score positively correlated with the cancellation of planned informal support, higher perceived risk for infection of COVID-19, difficulties in household finances, and lack of social support. Moreover, being younger, less wealthy, unemployed, and without a partner showed a significantly higher possibility of having a score above the cutoff.

**Conclusions:**

The present study found a high percentage of pregnant women with depressive symptoms. Notably, COVID-19-related variables, including perceived risk for the infection, fear of decreasing economic wealth, and social support, were significantly associated with depressive symptoms.

The coronavirus disease (COVID-19) pandemic has become an unprecedented global crisis. So far, we have not seen a decrease in mortality rate, and a cure or vaccine has not yet been developed. Social distancing has been a major preventive measure being implemented. While it may help to slow down the rate of infection and ease the fear of being infected with COVID-19, there may be unintended negative consequences due to social isolation and decreased economic activities. Because social support is recognized to have a buffering role in protecting pregnant women from stress during pregnancy,^1^ being isolated could make it even more difficult for pregnant women to cope with this crisis. Depression during pregnancy is known to increase the risk for adverse birth outcomes, and postnatal depression can have prolonged negative effects on both the mother and the child.^2,3^ Thus, assessing depressive symptoms in pregnant women and identifying factors associated with it are urgently needed for effective interventions to prevent negative psychological consequences of the COVID-19 pandemic.

## METHODS

This study used a cross-sectional study design. An online survey was conducted from May 31 to June 6, 2020, through 2 companies (Karadanote Inc. and baby calendar Inc.) providing services to pregnant and postpartum women through web applications and mailing services. The survey was conducted just after the state of emergency was lifted in all prefectures. We sent e-mails to users to solicit voluntary participation in our survey. Since users of their services include non-active and non-targeted groups, the exact percentage of response rate among our targets is indeterminate. We sent 607 458 e-mails, approximately 1.3% accessed our survey web page, of which approximately 74% (5650) replied. Respondents were from all prefectures, except Wakayama. Among the replies, 2105 were pregnant at the time of the survey, but 1777 responses were included in the study, after removing incomplete responses to the survey.

The present study was approved by the Research Ethics Committee of the University of Tsukuba (No. 2020-1). In order to protect respondents from having negative emotions triggered by answering our questionnaires due to the content of our questionnaires, the letter of request included a summary of the contents of our survey. Additionally, participants could withdraw from the survey at any time, and all questions were indicated as non-mandatory.

### Data Collection

We collected data from self-administered questionnaires. The Japanese version of the Edinburgh Postnatal Depression Scale (EPDS) was employed to measure depressive symptoms.^4^ A good internal consistency of the EPDS, which included 10 items, was shown with a Cronbach’s alpha of 0.87. The questionnaire also collected information about the perceived risk of COVID-19 infections, its negative consequences, unexpected experiences under the COVID-19 pandemic, as well as sociodemographic and economic information.

### Analysis

We first calculated the percentage of women with an EPDS score of ≥ 13 and factor scores of anhedonia (EPDS items 1 and 2), anxiety (EPDS items 3, 4, and 5), and depression (EPDS items 7, 8, and 9) for the first, second, and third trimesters, respectively. This cutoff score was validated by Usuda et al.^5^ A logistic regression analysis was performed by setting a dependent variable as 1: EPDS score of ≥ 13; 0: EPDS score of < 13 after adjusting for respondents’ sociodemographic and economic backgrounds (see Table 1 for the list of covariates and their definitions). Additionally, ordinary least squares regressions were performed to regress on each factor score. We included 3 COVID-19 pandemic-related variables: experiences during the COVID-19 pandemic, perceived risk, and place of residence. These variables were sorted into 5 groups based on the day the state of emergency was lifted. All statistical analyses were performed using Stata/MP 15.1 (StataCorp, College Station, TX).

**TABLE 1:** Summary Statistics

Variable	Percentage/Mean	SD	Min	Max
**Dependent Variable**				
Edinburgh Postnatal Depression Scale (EPDS) ≥ 13 (1: EPDS ≥ 13, 0: EPDS < 13)	17.0%	0.38	0	1
Anhedonia	0.72	1.11	0	6
Anxiety	3.68	2.28	0	9
Depression	1.82	2.05	0	9
**Independent Variables**				
COVID-19-related variables				
Experiences during COVID-19				
Changes of place of delivery (1: Yes, 0: No)	7.8%	0.27	0	1
Cancellation of parenting classes (1: Yes, 0: No)	79.3%	0.40	0	1
Prohibition of visitors at hospitals (before and after the delivery) (1: Yes, 0: No)	70.0%	0.46	0	1
Prohibition of an entry of a partner into delivery room (1: Yes, 0: No)	63.5%	0.48	0	1
Cancellation of planned informal support (1: Yes, 0: No)	12.6%	0.33	0	1
Cancellation of planned formal support (1: Yes, 0: No)	10.0%	0.30	0	1
Lifestyle changes with a partner working from home (1: Yes, 0: No)	20.0%	0.40	0	1
Perceived risk (COVID-19 related): How respondents think they will be directly affected by following things in the next 6 months.				
Financial difficulties (1: Not at all likely – 7: Very likely)	3.86	1.90	1	7
COVID-19 infection (1: Not at all likely – 7: Very likely)	4.09	1.55	1	7
Not receiving formal childcare support (1: Not at all likely – 7: Very likely)	4.39	1.84	1	7
Not receiving informal childcare support (1: Not at all likely – 7: Very likely)	3.40	1.93	1	7
Place of residence: Mothers were grouped into the following categories based on the day the state of emergency ended. The longest duration was Tokyo/Kanagawa/Saitama/Chiba followed by Hokkaido, Osaka/Kyoto/Hyogo, and Aichi/Fukuoka.				
Tokyo/Kanagawa/Saitama/Chiba (1: Tokyo/Kanagawa/Saitama/Chiba, 0: Other)	35.23%	0.48	0	1
Osaka/Kyoto/Hyogo (1: Osaka/Kyoto/Hyogo, 0: Other)	16.77%	0.37	0	1
Aichi/Fukuoka (1: Aichi/Fukuoka, 0: Other)	12.27%	0.33	0	1
Hokkaido (1: Hokkaido, 0: Other)	3.10%	0.17	0	1
Other prefectures (1: Other prefectures, 0: Other)	32.64%	0.47	0	1
Respondents’ sociodemographic, socioeconomic information.				
Age of the respondents (mother’s age)				
Age < 25 years (1: age < 25 years, 0: Other)	5.35%	0.23	0	1
Age 25-29 years (1: 25 years ≤ age < 30, 0: Other)	29.21%	0.45	0	1
Age 30-34 years (1: 30 years ≤ age < 35, 0: Other)	37.20%	0.48	0	1
Age ≥ 35 years (1: age ≥ 35 years, 0: Other)	28.25%	0.45	0	1
Nulliparous (1: Nulliparous, 0: Primiparous or multiparous)	65.17%	0.48	0	1
Pregnancy stage				
First trimester	13.22%	0.34	0	1
Second trimester	41.70%	0.49	0	1
Third trimester	45.08%	0.50	0	1
Number of children	0.5273	0.93	0	9
Household annual total income including tax				
Lower income group (1: < 5 million yen 0: ≥ 5 million yen)				
	44.46%	0.50	0	1
Working/full-time housewife status				
Full-time employed worker (1: Full-time employed worker, 0: Other)	48.68%	0.50	0	1
Full-time housewife/student (1: Full-time housewife/student, 0: Other)	27.07%	0.44	0	1
Working for family business/freelancer (1: Working for family business/freelancer, 0: Other)	2.98%	0.17	0	1
Contracted/part-time employed worker (1: Contracted/part-time employed worker, 0: Other)	19.75%	0.40	0	1
Unemployed (under job search) (1: Unemployed, 0: Other)	1.52%	0.12	0	1
Highest educational attainment				
Educational attainment less than 16 years (1: < 16 years, 0: ≥ 16 years)	19.30%	0.39	0	1
Marital status				
Married (1: Married, 0: Other)	96.40%	0.19	0	1
Unmarried (1: Unmarried, 0: Other)	2.76%	0.16	0	1
Divorced/widowed (1: Divorced/widowed, 0: Other)	0.84%	0.09	0	1
Number of family members who have been involved in childcare on a daily basis in the past 2 months (we have specified the period as the past 2 months since it was the time that most people have faced closure of childcare support center/nursery/kindergarten)	2.08	1.79	0	11

n = 1777

## RESULTS

The point prevalence of pregnant women with an EPDS score of ≥ 13 was 17% overall and 20%, 17.9%, and 15.2% for the first (n = 235), second (n = 741), and third trimester (n = 801), respectively, although no statistical significance was observed by the Kruskal–Wallis test. Breaking down to factor scores, the mean scores were 0.73, 3.68, 1.82 for anhedonia, anxiety, and depression, respectively.

In relation to COVID-19, we asked respondents whether they had experienced or were informed about: changes in place of delivery; cancellation of parenting classes; prohibition of visitors at hospitals; prohibition of an entry of a partner into a delivery room; cancellation of planned informal support; and cancellation of planned formal support. Results indicate nearly 80% of respondents experienced or were informed about the cancellation of parenting classes, showing the highest percentage, followed by prohibition of visitors at hospitals (70.0%), and prohibition of a partner into a delivery room (63.5%).

In terms of perceived risk, respondents were asked: “How do you think you will be directly affected by the following aspects in the next 6 months.” Response options ranged from 1: “Not at all likely” to 7: “Very likely.” The average score showed that pregnant women thought that the highest risk was in not being able to receive formal childcare support (4.39), followed by COVID-19 infection (4.09), financial difficulties (3.86), and not being able to receive informal childcare support (3.40).

Table 2 shows the results of regression analyses. For brevity, it shows only the variables statistically associated with depressive symptoms. For experiences of unexpected changes, respondents who have experienced cancellation of planned informal support had a higher risk of having depressive symptoms (OR, 1.79; 95% CI: 1.22–2.61). For perceived risk variables, pregnant women who experience greater risk of financial difficulties, COVID-19 infection, and not being able to receive informal childcare support are independently associated with an EPDS score of ≥ 13 (OR, 1.19; 95% CI: 1.10–1.28, OR, 1.13; CI: 1.02–1.25, and OR, 1.13; 95% CI: 1.03–1.23, respectively). As for sociodemographic/economic variables, younger (age less than 25 years old) (OR, 1.80; 95% CI: 1.02–3.18 in reference to age 35 or older), lower income (OR, 1.47; 95% CI: 1.09–1.97 in reference to higher income group of 5 million yen or more per annum), full-time housewife/student, and unemployed pregnant women (OR, 1.43; 95% CI: 1.03–1.98, and OR, 2.51; 95% CI: 1.08–5.82, respectively, in reference to full-time worker). Compared with married women, women without a partner (never married, divorced, or widowed) were more likely to show higher probability of having EPDS score of 13 or above (OR, 2.16; 95% CI: 1.11–4.18, and OR, 3.43; 95% CI: 1.14–10.36, respectively).

**TABLE 2:** Results of Regression Analyses

	EPDS ≥ 13	Anhedonia	Anxiety	Depression
	Odds Ratio [95% CI]	Coefficients [95% Cl]	Coefficients [95% CI]	Coefficients [95% CI]
**Experiences during COVID-19**				
Cancellation of planned informal support	1.79**	0.29	0.23	0.40*
	[1.22-2.61]	[0.09-0.49]	[-0.10-0.55]	[0.05-0.75]
**Perceived risk (COVID-19 related)**				
Financial difficulties	1.19***	0.07***	0.14***	0.14***
	[1.10-1.28]	[0.04-0.09]	[0.08-0.21]	[0.08-0.20]
COVID-19 infection	1.13*	0.04*	0.18***	0.07
	[1.02-1.25]	[0.01-0.08]	[0.09-0.26]	[-0.00-0.14]
Not receiving informal childcare support	1.13**	0.05**	0.15***	0.12***
	[1.03-1.23]	[0.02-0.09]	[0.08-0.22]	[0.06-0.19]
Respondents’ sociodemographic, socioeconomic information				
Age of the respondents (Ref: Age ≥ 35 years)				
Age < 25 years	1.80*	-0.12	1.03***	0.64*
Lower income group (Ref: ≥ 5 million yen)	1.47*	0.08	0.36**	0.33**
	[1.09-1.97]	[-0.03-0.18]	[0.14-0.58]	[0.13-0.54]
Working/full-time housewife status (Ref: Full-time employed worker)				
Full-time housewife/student	1.43*	0.01	0.39**	0.16
	[1.03-1.98]	[-0.17-0.14]	[0.13-0.64]	[-0.07-0.40]
Unemployed (under job search)	2.51*	0.68*	0.02	0.64
	[1.08-5.82]	[0.15-1.20]	[-1.03-1.08]	[-0.40-1.67]
Marital status (Ref: Married)				
Unmarried	2.16*	0.20	-0.04	1.20**
	[1.11-4.18]	[-0.15-0.56]	[-0.68-0.59]	[0.48-1.93]
Divorced/widowed	3.43*	1.14*	1.82**	2.99***
	[1.14-10.36]	[0.28-2.01]	[0.44-3.19]	[1.46-4.51]
Constant	0.02***	0.289	1.033	0.157
	Log likelihood = -735.215	R^2^ = 0.101	R^2^ = 0.129	R^2^ = 0.131
	LR chi2(31) = 149.50***	F (31.1745) = 4.70***	F (31.1745) = 8.87***	F (31.1745) = 7.25***

*Notes*: ***P < 0.001, **P < 0.01, *P < 0.05; number of observations: 1777.

Looking into further analyses on factor scores regression, some variables were correlated with certain factors, whereas others were associated with all factors, and magnitudes of associations differed. Cancellation of planned informal support increased the depression score by 0.4 points. Because the average score for depression was 1.82 points, this result translated the depression score to 2.22 points when a pregnant woman faced cancellation of planned informal support. For perceived risk, a 1-point increase in the risk score of financial difficulties leads to an increase in factor scores of anhedonia, anxiety, and depression by 0.07, 0.14, and 0.14 points, respectively. The statistical significance and coefficients of perceived risk for not receiving informal childcare support are similar. For COVID-19 infection risk, a 1-point increase in risk score leads to a 0.18- and 0.04-point increase in anxiety and anhedonia scores, respectively. Younger age and lower income increased anxiety and depression scores. For these variables, anxiety scores were affected more severely than depression scores as coefficients showed that a younger age (less than 25 years old) was likely to have a 1.03-point higher score, and the lower income group was likely to have a 0.36-point higher score in anxiety. Being a full-time housewife/student was associated with an increased score of anxiety by 0.39 points while being unemployed, related to higher anhedonia scores (coefficient, 0.68). Unmarried women are likely to have a higher score of depression by 1.20 points. Divorced/widowed women are likely to have higher scores for all factors with the strongest impact on the depression score, indicating that they have a 3-point higher score in depression in comparison with married women.

## DISCUSSION

Reports from China, Turkey, and Canada have presented elevated depression and anxiety among pregnant women during the COVID-19 pandemic, as the percentages of EPDS ≥ 13 were 29.6%, 35.4%, and 37%, respectively.^6-8^ Our study demonstrated that Japan is no exception. A study conducted in 2012–2013 by Takehara et al.^9^ showed that the total EPDS score and factor scores of anxiety, anhedonia, and depression in pregnant women at 20 weeks were 3.58, 2.00, 0.16, and 0.76, respectively. Although rigorous comparisons are not possible, the socioeconomic background of their study subjects is similar to ours, except that educational attainment is higher for this study.

From our regression analyses, in addition to fear of the infection of COVID-19, unintended negative consequences due to social distancing, including travel restriction and economic downturn, were found to have a significant association with depressive symptoms. Factor score analyses showed some important insights. For instance, perceived risk for COVID-19 infection increased the scores of anxiety and anhedonia, whereas perceived risk for financial difficulties and not receiving informal childcare support were affecting all factors, including depression. This suggests that depressive symptoms may last even if the fear of COVID-19 infection waned, unless economic stability and sufficient childcare are provided. Moreover, location of residence did not show any statistical association, indicating that the influence of COVID-19 was not limited to areas with higher reported positive cases. Furthermore, the negative effect of unintended outcomes of social isolation, or travel restrictions, causing economic uncertainty and insufficient childcare support had a strong impact on the mental well-being of women.

Sociodemographic characteristics of the high-risk group were clearly shown – younger, lower income, unemployed/housewife/student – which implies no cash income by herself, and women without a partner who have less monetary wealth, and informal childcare support in general. It is important to note that odds ratios and coefficients of factor scores indicated women without a partner were particularly at risk; divorced/widowed women faced 3.43 times higher risk, and never-married women had 2.16 times higher risk of having an EPDS score ≥ 13 than married women. When examining factor scores, divorced/widowed women were found to be facing anhedonia, anxiety, and severe depression. In particular, considering that the maximum points of depression are 9, a 3-point increase is a remarkably large increase.

Considering the growing prevalence of prenatal depression and its prolonged adverse effects, urgent interventions are needed in clinical care and social policy. Although some municipalities have been providing additional financial support for households with children, there is, to the author’s knowledge, currently no social policy intervention for pregnant women. Infection is likely to be higher among pregnant women due to a weakened immune system and unknown fetal effects.

As for the labor policy, early paid maternity leave or special paid holidays for pregnant women could reduce unnecessary human contact. Indeed, in our observation sample, among those who had a job, about 40% commuted to work, 22% worked from home, and 13% worked alternately from home or office. Approximately 5% of pregnant women had quit their jobs due to fear of contracting the COVID-19 infection.

As for social policy, additional financial support is necessary. Contract workers or part-time workers lose their income if they decide not to work. This would cause financial difficulties, in turn leading to limited options in terms of health care. Additionally, prenatal care and delivery are not free. Although (usually generous) subsidies are provided, they may not be adequate during the current pandemic. Prenatal check-ups are indispensable; the pandemic increases the risk of prenatal depression, and it is essential to treat it appropriately. With the cancellation of parenting classes or other pregnancy support activities, prenatal check-ups are probably the only place where pregnant women can talk face-to-face about their concerns or fears. Thus, financial support is essential to ensure that pregnant women attend prenatal check-ups.

As there is no guarantee that the COVID-19 pandemic will end soon, women who are currently pregnant will most likely give birth during this pandemic. Thus, collaboration during clinical care is becoming increasingly important among midwives, obstetricians, pediatricians, psychiatrists, and counselors, especially if a pregnant woman exhibits signs of depression. Moreover, cooperation between health professionals and social workers or community workers is required to support pregnant mothers during the current pandemic to ensure a healthy pregnancy and delivery. This is because personal attributes as well as COVID-19-related fears, the consequences of social distancing, and economic uncertainty increase the possibility of depression.

### Study Limitation

The present study has some limitations. First, being an online survey, we can only approximate the response rate. Given that online surveys are based on voluntary participation, there is a chance that those who have severe depressive symptoms are not able to access or complete this survey. Another limitation is that our survey data did not include information on mothers’ past psychiatric episodes, past pregnancy experiences, or current physical health conditions.

## CONCLUSION

The present study found a high prevalence of depression among pregnant women due to the COVID-19 pandemic. Social policy intervention as well as cooperation within clinical care, between health care professionals, and social/community workers are urgently required.
